# Transfusion-Dependent Anaemia: An Overlooked Complication of Paraoesophageal Hernias

**DOI:** 10.1155/2014/479240

**Published:** 2014-07-24

**Authors:** Richard J. E. Skipworth, Ralph F. Staerkle, Steven Leibman, Garett S. Smith

**Affiliations:** Department of Gastrointestinal Surgery, Royal North Shore Hospital, Reserve Road, St Leonards, NSW 2065, Australia

## Abstract

*Introduction*. A paraoesophageal hernia (PH) may be one reason for iron-deficiency anaemia (IDA) but is often overlooked as a cause. We aimed to assess the incidence and resolution of transfusion-dependent IDA in patients presenting for hiatal hernia surgery. *Methods*. We analysed a prospective database of patients undergoing laparoscopic hiatal repair in order to identify patients with severe IDA requiring red cell/iron transfusion. *Results*. Of 138 patients with PH managed over a 4-year period, 7 patients (5.1%; M : F 2 : 5; median age 62 yrs (range 57–82)) with IDA requiring red cell/iron transfusion were identified. Preoperatively, 3/7 patients underwent repetitive and unnecessary diagnostic endoscopic investigations prior to surgery. Only 2/7 ever demonstrated gastric mucosal erosions (Cameron ulcers). All patients were cured from anaemia postoperatively. *Discussion*. PH is an important differential diagnosis in patients with IDA, even those with marked anaemia and no endoscopically identifiable mucosal lesions. Early recognition can avoid unnecessary additional diagnostic endoscopies. Laparoscopic repair is associated with low morbidity and results in resolution of anaemia.

## 1. Introduction

The three main causes of anaemia are blood loss, lack of red blood cell production, and high rates of red blood cell destruction. Blood loss is the most common cause of anaemia, especially iron-deficiency anaemia (IDA). Between 2 and 5% of adult men and postmenopausal women in the developed world have IDA [1, 2]. In premenopausal women, menstrual blood loss is the most common cause for IDA, but, in postmenopausal women and men, the commonest cause is blood loss from the gastrointestinal (GI) tract [3–6]. The most frequent causes of occult GI blood loss are nonsteroidal anti-inflammatory drugs, colonic cancer/polyp, gastric cancer, angiodysplasia, Crohn's disease, and ulcerative colitis [7]. Therefore, an upper and lower endoscopy is an important and necessary examination in the assessment of anaemic patients.

Apart from the more usual causes of IDA, there are also less frequent and often overlooked causes of GI blood loss, such as paraoesophageal herniae (PH). The prevalence of IDA in patients with large PH is between 6 and 37% [8–11], a clinical correlation first established by Segal in 1931 [12]. However, the exact cause of this association is not fully elucidated. In 1986, Cameron and Higgins described the presence of linear gastric erosions at the waist of the hernia (so called Cameron lesions), which can cause chronic blood loss [13]. Although the association between Cameron lesions and hiatal hernias is well described [14, 15], the pathophysiological mechanisms underlying these lesions remain unclear. Suggested explanations include mechanical trauma, reflux [13], and ischaemia [16]. Patient age and sex may also be contributory factors [17]. However, these lesions do not fully explain IDA, as only 1/3 or less of anaemic patients with a large PH might have Cameron lesions at endoscopy [9, 18].

Although IDA is a recognised complication of hiatus hernia, there are few published data regarding the incidence and outcome of severe, transfusion-dependent IDA in patients with PH (as opposed to mild anaemia not requiring treatment). The aim of this study was to specifically identify such patients and to assess resolution of the anaemia after laparoscopic repair of the hiatal hernia.

## 2. Materials and Methods

### 2.1. Patients

Between August 2006 and November 2010, data were collected prospectively on all patients undergoing laparoscopic repair of PH. Patients were followed up routinely with clinical assessment and blood tests to assess the resolution of anaemia and other hernia-related symptoms. Patients with severe IDA (haemoglobin < 8 g/dL) [19] requiring red cell transfusion or iron infusion were retrospectively identified from the prospective database.

### 2.2. Standard Surgical Technique

All procedures were completed laparoscopically with the patient in the modified lithotomy position and the surgeon standing between the patient's legs. The hernia sac was dissected away from its mediastinal attachments and the oesophagus dissected to ensure adequate intra-abdominal length. After the crural pillars were approximated with 0 Ethibond sutures (Ethicon, Johnson & Johnson, Cincinnati, Ohio, USA), TiMesh titanized polypropylene mesh (Biomed Biologics, Nuremberg, Germany) was fixed to the crural muscle with laparoscopic helical screws (Protack 5 mm; Covidien, Mansfield, MA, USA). A posterior 270° (Toupet) fundoplication was then usually carried out.

## 3. Results

During the study period, 138 patients were surgically managed for PH. Seven patients (5.1%; M : F 2 : 5; median age 62 years (57–82)) were identified as having a transfusion-dependent (blood or iron) anaemia at surgical presentation. Amongst the group, the lowest recorded preoperative haemoglobin concentration was 38 g/L. The most common symptoms beside anaemia were chest pain (*n* = 4), shortness of breath (*n* = 3), regurgitation (*n* = 3), dysphagia (*n* = 1), and heartburn (*n* = 1). The median time duration between symptom onset and date of surgery was 12.0 months (range 3–120 months). Appropriately, all patients had undergone preoperative diagnostic upper GI endoscopy, 6/7 had undergone diagnostic colonoscopy, and 3/7 had undergone capsule endoscopy. However, as PH was not considered as an explanatory cause for severe, transfusion-dependent IDA or gastric ulceration, several patients waited significant lengths of time prior to being considered for surgery (in one case, 10 years). During these delays in surgical treatment, 2/7 patients underwent unnecessary repeated endoscopic investigations after initial tests had yielded negative findings and a further patient underwent a second upper GI endoscopy to check for resolution of gastric ulceration (Cameron lesion) before being referred for surgery. (Any follow-up endoscopy could have been performed at the time of surgery.) In total, these 3 patients underwent the following:

 Patient 1: 10 upper GI endoscopies, 10 colonoscopies, and 2 capsule endoscopies,
 Patient 2: 3 upper GI endoscopies and 3 colonoscopies
 Patient 3: 2 upper GI endoscopies.

Cameron lesions were historically identified in only 2 patients, but in 1 of these individuals the erosions had healed and disappeared prior to surgery (Patient 3). Apart from the PH identified endoscopically, no other mucosal abnormalities were identified within the GI tracts of these 7 patients to explain IDA. Haematological investigations also revealed no other potential causes of bleeding.

During preoperative investigation and management, patients received either red cell transfusions (*n* = 5) and/or iron infusions (*n* = 2). Another one of the patients was due to have a red cell transfusion but this did not occur prior to the date of operation. In those patients receiving blood, the total number of units of packed red cells transfused ranged from 2 to 36 (Patient 1 received bimonthly blood transfusions for 6 years prior to surgery). All patients underwent laparoscopic mesh repair of PH with a fundoplication procedure (6 posterior 270° Toupet, and 1 anterior 180° Dor procedure due to an inability to mobilise the fundus sufficiently for a posterior wrap). Postoperatively, there were no complications and no in-hospital or 30-day mortality. The median postoperative stay was 3.5 days (2–5 days). During a follow-up period of 1 year, the anaemia was resolved in all cases and no further transfusions were required.

## 4. Discussion

Paraoesophageal (or rolling) hiatal hernias may affect adults of any age and sex but are most common in elderly women. Symptoms are due to the mechanical effects of the hernia and are therefore often resistant to pharmacological treatment. In contrast to the more common sliding hiatal hernia, a PH can lead to volvulus with pain and obstructive symptoms or, as our series demonstrates, they can be associated with IDA, including severe, transfusion-dependent anaemia. In fact, severe anaemia appears to have a prevalence of 5% in these patients. Such anaemia may occur even in the apparent absence of Cameron lesions or obvious bleeding focal points at endoscopy. In our series, only 2 patients were ever noted to have gastric erosions. However, it is worth noting that, in 1 of these patients, the Cameron lesions resolved, suggesting either that occult bleeding is an intermittent phenomenon that is not always present at the time of endoscopy or, less likely, that bleeding lesions are missed at endoscopy. Nevertheless, other authors have found no correlation between Cameron lesions and visible GI blood loss [18] or haemoglobin levels [19]. An alternative explanation for IDA might be that large hiatus hernias interfere with the absorption and regulation of dietary iron.

It is obviously vital that all patients with IDA of unknown cause require upper GI endoscopy and colonoscopy. However, in our patient group, despite the severity of anaemia, patients experienced symptoms for a median duration of 1 year before being referred to surgical services for a hiatal repair. During this preoperative assessment stage, 3/7 patients underwent repeated endoscopic investigations and transfusions and were therefore unnecessarily exposed to the risk of procedure-related complications. Furthermore, the cost of such preventable interventions would not have been insignificant. Repeated upper GI endoscopy is appropriate in those requiring endoscopic treatment for acute life-threatening haemorrhage from upper GI ulceration but not in those with chronic IDA. It is therefore vitally important that physicians who assess patients with IDA are cognisant of the correlation with hiatus hernia, particularly in the absence of erosive pathology at endoscopy. Any failure to appreciate this association might represent a lack of awareness on the part of physicians and surgeons managing IDA. For example, a review of 7 core European and Australasian surgical textbooks reveals that only 3 textbooks make reference to hiatus hernias being a cause of bleeding or anaemia, and, in these books, bleeding is chiefly attributed to reflux oesophagitis [20–26]. Cameron lesions are described in only one of these textbooks, whereas PH as a cause of cryptogenic anaemia is not discussed in any of them. Gastrooesophageal reflux disease (GORD) and oesophagitis do frequently coexist with hiatal hernias, and therefore oesophagitis may well be the cause of anaemia in some patients. However, PH (with or without Cameron lesions) should still be considered as an aetiological factor in severe IDA.

In our series, laparoscopic PH repair cured all patients of severe anaemia during a median follow-up period of 12 months. Hayden and Jamieson also included 3 patients with transfusion-dependent anaemia in their series of 11 patients with PH and IDA [27]. They also demonstrated resolution of patient anaemia during a median follow-up of 25 months [27]. Therefore, it would seem appropriate to offer surgical intervention to young, medically fit patients with PH and severe IDA with no other cause found on endoscopic investigation. Such an approach would also prevent the foregut and mechanical complications of PH, symptoms for which some authors would recommend routine surgical repair in any case [28]. This recommendation contrasts with those of Panzuto et al., who demonstrated in a randomised control trial that proton pump inhibitor (PPI) monotherapy was as effective as surgery with PPI at resolving IDA in association with large hiatal hernia [9]. However, this study did not involve pure cohorts of patients with severe, transfusion dependent anaemia but also included individuals with mild anaemia. Many patients with PH are elderly and frail and thus a nonoperative approach might be appropriate in this group. Elderly patient age alone remains only a relative contraindication to surgery, however, as we have shown previously that laparoscopic repair is associated with acceptable rates of morbidity and a significant improvement in quality of life in such individuals [29].

## 5. Conclusion

In summary, we have identified a small but significant number of patients with severe IDA in association with PH, often in the absence of endoscopically identifiable Cameron lesions. Early recognition of the fact that PH may be an aetiological factor in severe IDA will mitigate the likelihood of prolonged transfusion dependency and fruitless, repeated GI tract investigations as laparoscopic repair of PH resolves IDA.
